# Genome-wide association study confirm major QTL for backfat fatty acid composition on SSC14 in Duroc pigs

**DOI:** 10.1186/s12864-017-3752-0

**Published:** 2017-05-11

**Authors:** Maren van Son, Eli Gjerlaug Enger, Harald Grove, Roger Ros-Freixedes, Matthew P. Kent, Sigbjørn Lien, Eli Grindflek

**Affiliations:** 1 Norsvin SA, Storhamargata 44, , 2317 Hamar, Norway; 2 Centre for Integrative Genetics (CIGENE), Department for Animal and Aquacultural Sciences, Norwegian University of Life Sciences, P. O. Box 5003, 1432 Ås, Norway; 3 Departament de Ciència Animal, Universitat de Lleida-Agrotecnio Center, 191 Av Alcalde Rovira Roure, 25198 Lleida, Catalonia Spain; 4 Present address: The Roslin Institute and Royal (Dick) School of Veterinary Studies, The University of Edinburgh, EH25 9RG Midlothian, Scotland UK

**Keywords:** Fatty acid composition, GWAS, QTL mapping, Fine mapping, Pigs

## Abstract

**Background:**

Fatty acid composition contributes importantly to meat quality and is essential to the nutritional value of the meat. Identification of genetic factors underlying levels of fatty acids can be used to breed for pigs with healthier meat. The aim of this study was to conduct genome-wide association studies (GWAS) to identify QTL regions affecting fatty acid composition in backfat from the pig breeds Duroc and Landrace.

**Results:**

Using data from the Axiom porcine 660 K array, we performed GWAS on 454 Duroc and 659 Landrace boars for fatty acid phenotypes measured by near-infrared spectroscopy (NIRS) technology (C16:0, C16:1n-7, C18:0, C18:1n-9, C18:2n-6, C18:3n-3, total saturated fatty acids, monounsaturated fatty acids and polyunsaturated fatty acids). Two QTL regions on SSC4 and SSC14 were identified in Duroc for the *de novo* synthesized fatty acids traits, whereas one QTL on SSC8 was detected in Landrace for C16:1n-7. The QTL region on SSC14 has been reported in previous studies and a putative causative mutation has been suggested in the promoter region of the *SCD* gene. Whole genome re-sequencing data was used for genotype imputation and to fine map the SSC14 QTL region in Norwegian Duroc. This effort confirms the location of the QTL on this chromosome as well as suggesting other putative candidate genes in the region. The most significant single nucleotide polymorphisms (SNPs) located on SSC14 explain between 55 and 76% of the genetic variance and between 27 and 54% of the phenotypic variance for the *de novo* synthesized fatty acid traits in Norwegian Duroc. For the QTL region on SSC8 in Landrace, the most significant SNP explained 19% of the genetic variance and 5% of the phenotypic variance for C16:1n-7.

**Conclusions:**

This study confirms a major QTL affecting fatty acid composition on SSC14 in Duroc, which can be used in genetic selection to increase the level of fatty acid desaturation. The SSC14 QTL was not segregating in the Landrace population, but another QTL on SSC8 affecting C16:1n-7 was identified and might be used to increase the level of desaturation in meat products from this breed.

**Electronic supplementary material:**

The online version of this article (doi:10.1186/s12864-017-3752-0) contains supplementary material, which is available to authorized users.

## Background

The fatty acid composition of meat is important for its nutritional properties influencing human health, while also affecting the technical and sensory quality of meat products [1]. Fatty acids may be divided into two groups based on whether they are derived directly from the diet (essential) or whether they can be synthesized *de novo* through lipogenesis (non-essential). Fatty acids up to 16 carbons in length are synthesized *de novo* and some are, together with fatty acids from the diet, further elongated into fatty acids with more than 18 carbons in length. These saturated fatty acids (SFA; C16:0 and C18:0) can then be desaturated to monounsaturated fatty acids (MUFA; C16:1n-7 and C18:1n-9). Polyunsaturated fatty acids (PUFA), with two or three double bounds, are obtained from the diet [1].

Fatty acid composition has received attention due to public health concerns related to evidence that saturated fat can increase the amount of cholesterol in our blood, which may in turn increase the risk of heart disease and stroke. Healthier fat can be produced by increasing the levels of MUFA and PUFA, lowering levels of SFA and decreasing the ratio of n-6/n-3 PUFA [1–4]. The healthiness of the different types of fat is however debated, and some studies show that SFA is not as dangerous as previously claimed [5, 6]. Fatty acid composition is also important for meat quality, and high levels of PUFA negatively impacts meat quality traits such as oxidative stability and flavor [2]. Increasing the content of the MUFA C18:1n-9, however, could improve both organoleptic and technological qualities as well as nutritional properties [7, 8].

Previous studies in Landrace and Duroc have obtained high heritabilities for the fatty acids C16:0, C16:1n-7, C18:0, C18:1n-9, C18:2n-6 and C18:3n-3 [9]. The heritability estimates, ranging from 0.25 to 0.67, are in agreement with other studies [10, 11], suggesting that breeding pigs for favorable fatty acid composition is possible.

Numerous studies in different pig populations have identified quantitative trait loci (QTLs) for fatty acid composition in pig [12–25]. Significant QTLs have been identified on all the pig chromosomes and some are shared across breeds, populations and tissues or muscle groups. Candidate gene studies have been conducted to possibly identify functional mutations underlying the differences in fatty acid composition. On SSC8, promising results have been found for *microsomal triglyceride transfer protein* (*MTTP*), which is involved in lipoprotein assembly [26], and *ELOVL fatty acid elongase 6* (*ELOVL6*), which catalyzes the elongation of C12-16 fatty acids to C18 [17, 27]. On SSC14, the *stearoyl-CoA desaturase* (*SCD*) gene strikes out as a potent positional candidate gene for fat desaturation. The gene has been investigated in several studies [28–32] and a putative causal variant in the promoter region has been identified [28].

The aim of this study was to detect genomic regions controlling fatty acid composition in backfat from the Norwegian Duroc and Landrace breeds. For this purpose, six different fatty acid were measured in backfat from boars at a laboratory by NIRS technology, specifically C16:0, C16:1n-7, C18:0, C18:1n-9, C18:2n-6, C18:3n-3. Moreover, total SFA, MUFA and PUFA were included in the analyses. Boars were genotyped using the Axiom porcine 660 K SNP array (Affymetrix Inc.) and a GWAS was conducted. Whole genome re-sequencing data from related boars was used for subsequent fine mapping.

## Methods

### Animals

Animals from three groups of boars were included in this study. Group 1 was composed of 454 Duroc and 659 Landrace boars born in 2011 and 2012 from Norsvin’s boar testing station. Animals were kept in single-breed groups of 12 pigs per pen and fed ad libitum on conventional concentrates. The diet contained 161 and 136 g digestible protein, and 9.68 and 9.50 MJ net energy/kg before and after 50 kg live weight, respectively, with one month of mixing the two feeds to facilitate the feed change. Major feedstuff compounds were barley, oats, peas, soy meal extract and rendering (animal) fat. Average fat percentage of the feeds was 2.6%, with the following fatty acid profile (in percentage of total fatty acids): C16:0, 22.2%; C16:1n-7, 1.0%; C18:0, 8.9%; C18:1n-9, 24.2%; C18:2n-6, 35.4%; C18:3n-3, 4.1%; total SFA, 33.2%; total MUFA, 26.3%; and total PUFA, 39.8%. The average start and end weight of the pigs for the test was 35 and 120 kg live weight, respectively. Slaughter weight can vary some as the boars are waiting for selection or not, and this was corrected for using a simple generalized linear model (GLM). The boars in this test were all selection candidates to be elite boars for artificial insemination (AI). Non-selected boars goes to slaughter and are available for meat quality assessment, including the ones in this study. The sacrifice procedure is described in more detail in Gjerlaug-Enger et al. [33] and was in compliance with national guidelines. For the animals in this study, phenotypes in terms of fatty acid composition and genotypes from the Illumina porcine 60 K SNP chip (Illumina) were available.

Group 2 animals included 140 Duroc and 207 Landrace boars that were genotyped using the Axiom porcine 660 K array. These animals were frequently used AI boars between 2010 and 2015 and were close relatives to Group 1 animals. 60 K genotypes were also available from all the Group 2 animals. Additional 60 K genotypes were available for several thousand relatives making imputation of genotypes from 60 to 660 K feasible.

Whole genome re-sequencing data was available from Group 3 animals constituting 23 Duroc and 24 Landrace boars. These boars were frequently used as AI boars during the years 2010 to 2013 and overlap with Group 2 animals (6/10 of the Duroc/Landrace Group 3 animals were also Group 2 animals). 60 K genotypes were also available for the Group 3 animals.

### Fatty acid measurements

Subcutaneous fat samples were collected from half-sib tested animals after slaughter at Animalia’s pilot plant (the Norwegian Meat and Poultry Research Centre, Oslo, Norway). The samples were collected from the area by the last thoracic vertebrae, stored at −40 °C and thereafter prepared at the BioBank AS (Hamar, Norway). Before analyses, they were thawed and minced, followed by isolation of total lipids using a microwave fat melting technique [34]. A XDS near-infrared rapid content analyzer (FOSS NIRSystems, Hillerød, Denmark) was used to obtain transflection spectra of the total lipids from the samples. The fatty acids measured were palmitic (C16:0), palmitoleic (C16:1n-7), stearic (C18:0), oleic (C18:1n-9), linoleic (C18:2n-6) and α-linolenic (C18:3n-3). Each was measured as percentage of total fatty acids in backfat and expressed as grams/100 grams. Low percentage fatty acids were not included due to concerns over accuracy for low concentration measurements. In total, the above-mentioned fatty acids accounted for 95% of the total fatty acid content. Finally, total percentages of SFA, MUFA and PUFA were measured according to methods and calibration curves described in previous publications [9, 35].

### Genotyping

Genomic DNA was extracted from ear biopsies using BioSprint DNA Kit (Qiagen, Hilden, Germany). DNA concentration and quality was measured using a NanoDrop ND-1000 spectrophotometer (NanoDrop Technologies, DE, USA). Genome wide SNP genotyping was performed using either the Axiom porcine 660 K array from Affymetrix (Affymetrix Inc., Santa Clara, CA, USA) [36], which contains assays for 658,692 SNPs, or using the Illumina porcine 60 K SNP chip containing 62,163 SNPs (Illumina, San Diego, USA) [37]. For the Affymetrix array, genotypes were assigned using Axiom Analysis Suite following the best practices protocol recommendations. Genotypes from the Illumina array were generated using GenomeStudio software. SNPs were filtered based on call rate > 0.97 and minor allele frequency (MAF) > 0.01. All SNP positions were based on *Sscrofa* genome build 10.2 [38].

### Imputation from 60 K to 660 K

After removing 660 K SNPs not passing quality scores, we were left with 398,809 SNPs for 140 Duroc boars and 413,094 SNPs for 207 Landrace boars. The 5855 Duroc and 10,976 Landrace pigs genotyped on the 60 K SNP chip were imputed to 660 K using the available 660 K genotyped pigs as reference. The number of filtered, high quality 60 K SNPs overlapping with 660 K used for this imputation was 29,433 for Duroc and 30,971 for Landrace. SNPs that were shared between the arrays were checked for matching genotypes and allele frequencies. The imputation was performed separately for each breed. The software used was FImpute v.2.2 [39] and we used default settings together with the complete pedigree as additional information.

### Genome wide association analyses

GWAS analyses were run using the R v.3.2.4 package GenABEL v.1.8-0 [40, 41]. Phenotypes included the six fatty acids and three combined traits described above and they were pre-corrected for day of slaughter. For each trait, a test was run using the polygenic function, which combines the Family Based Score Test for Association (FASTA) with a kinship matrix of relatedness estimated from genotyped SNPs using the identity-by-state function. The *p*-values were corrected for genomic control by dividing the observed test statistic by a calculated genomic inflation factor, and a *p*-value of 1.0^−6^ was required for genome-wide significance. Manhattan plots were generated using the R package qqman v.0.1.2 [41, 42]. Genetic parameters were estimated using ASReml v.3.0 [43] by fitting SNP as a fixed effect and animal ID as a random effect. The fraction of genetic and phenotypic variance explained by each SNP for each phenotype was calculated as 2p(1-p)α^2^, divided by the additive genetic variance and phenotypic variance, respectively [44]. Here p is the frequency of the A allele in a SNP with the two alleles A and a, and α is the allele substitution effect. The Haploview software v.4.2 [45] was used with phased data to calculate linkage disequilibrium (LD) as expressed by r^2^.

### Sequencing data analyses

Genomic DNA from 23 Duroc and 24 Landrace boars was extracted from blood or semen samples using the MagAttract DNA Blood Midi M48 protocol on the Bio-Robot M48 (Qiagen, Hilden, Germany). Whole genome DNA sequencing was performed by a commercial provider using an Illumina GAII platform generating 2 × 100 bp reads according to manufactures’ instructions.

Reads were quality checked using FastQC v.0.10.1 (Babraham Bioinformatics, UK) and trimmed using Sickle with the options paired end, a length for trimming of 50 and otherwise default settings [46]. BWA-aln v.0.7.5 was used to align the reads to *Sscrofa* build 10.2 [47] using default settings. Duplicates were marked and files sorted with SAMtools v.0.1.19 [48] and SNPs in the most significant QTL region were detected using FreeBayes v.1.0.2 [49]. For the initial detection of putative variants, a minimum of 2x read coverage was set. The detected variants were filtered using VCFtools v.0.1.14 [50] and SAMtools bcftools v.1.3 [47] by the following criteria: minimum 2x read coverage for a new allele with both reference and alternate allele present on both strands, minimum quality score of 25, and a mapping quality of >10 for both alleles at a SNP position. A distance of at least 4 and 10 bp to the next insertion/deletion (indel) was applied for SNPs and indels, respectively, and variants with more than one unique non-reference allele were removed. The variants were also filtered on sequencing depth because such variants are likely to be located in duplicated regions and be the result of misalignment. The Ensembl Variant Effect Predictor (VEP) software was applied to predict the effect of the detected SNPs [51]. The whole genome re-sequencing generated 10.1 billion paired-end reads with coverage ranging from 9-17X across the entire genome. A total of 48,346 variants were detected in the 8 Mb QTL region in the Duroc pigs. After filtering, 18,252 SNPs were left in the reference panel for imputation.

### Imputation from 660 K to sequence

Genotype likelihoods outputted from FreeBayes were used to impute sporadic missing genotypes in the sequenced animals (reference panel) using Beagle v.4.1 [52, 53]. The same software was then used to phase the reference panel. Prior to imputation, the target panel (660 K) was compared to the reference panel using conform-gt [53] to exclude target variants without a corresponding reference panel record and to adjust target records to match the chromosome strand and allele order in the reference panel. The 660 K panel genotypes within the QTL region were imputed to the sequence based genotypes of a population of 5996 Duroc animals. After imputation, SNPs with a MAF < 0.01 were removed before further analyses. Conform-gt removed 9 of the 660 K SNPs due to unknown strand issues and 104 because they were not in the reference panel, leaving 1151 of the 660 K SNPs in the region for imputation. They were combined with the 18,252 SNPs in the reference panel and after filtering for MAF < 0.01, 13,565 SNPs were left for sequence based association analyses and representing one SNP every 590 bp on average. The newly detected and filtered SNPs have been submitted to dbSNP [54].

### Sequence based association analyses

Sequence based association analyses were run in ASReml fitting each SNP as a fixed effect in the model. The phenotypes were the same as in the GWAS analyses, and a pedigree-based relationship matrix was fitted. The *p*-values were corrected for multiple testing using the Bonferroni correction method as implemented in the stats package v.3.2.4 in R v.3.2.4 [41].

## Results

**Fig. 1:**
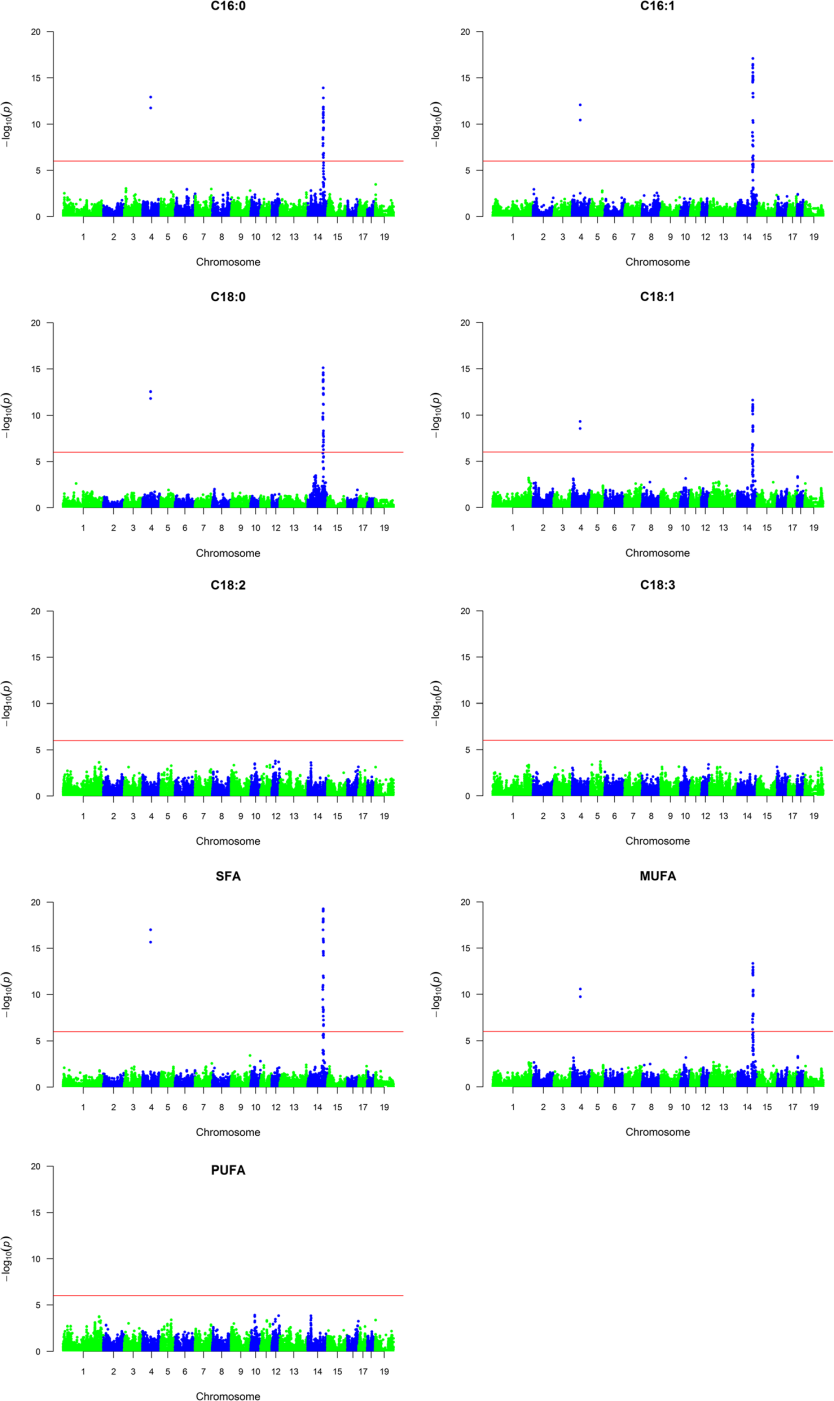
Manhattan plots showing genome-wide association results for fatty acid composition in Duroc. SNPs are plotted on the x-axis according to their position on each chromosome against statistical association with these traits on the y-axis. The *horizontal line* indicates genome-wide significance (*p* < 1.0^−6^)

**Fig. 2:**
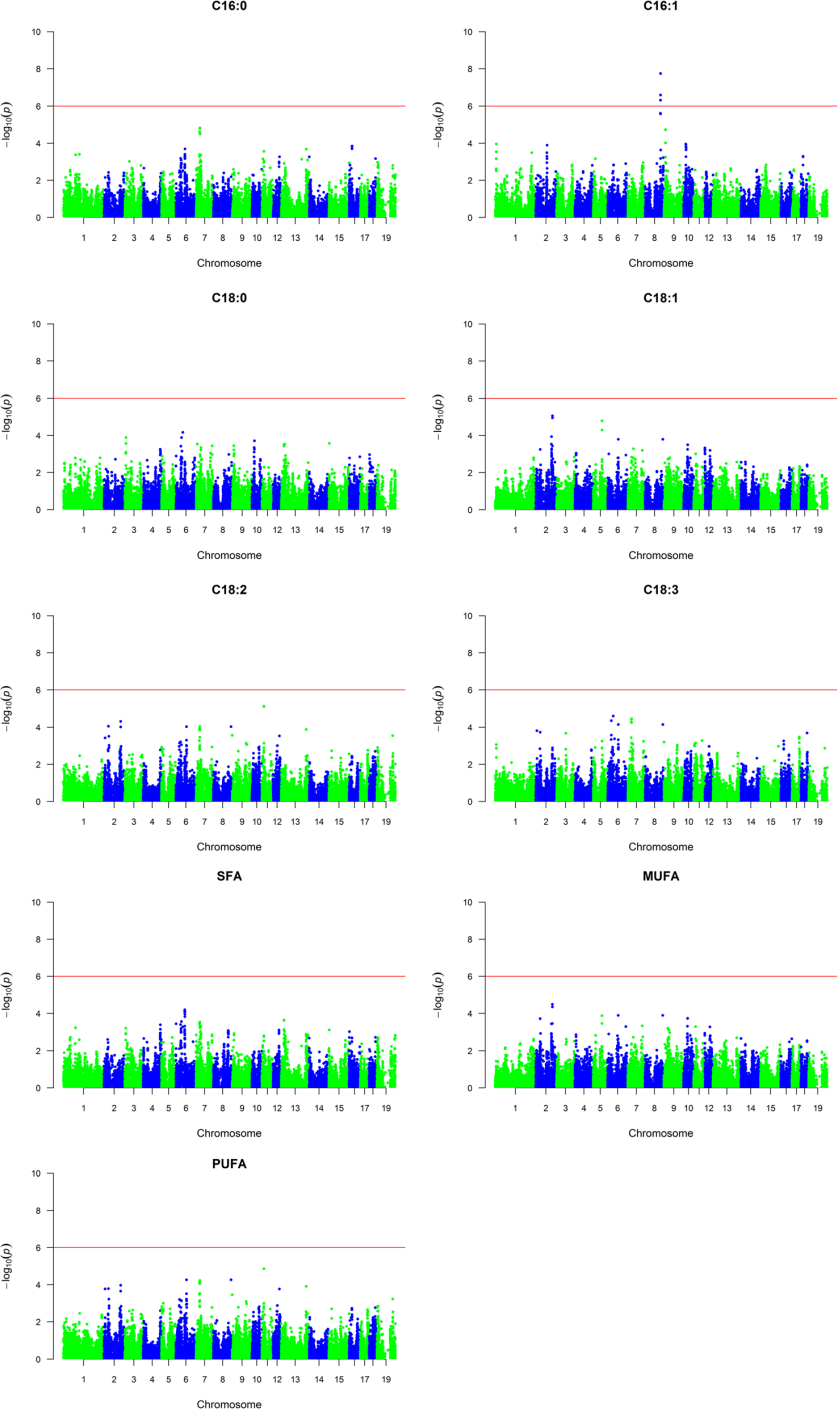
Manhattan plots showing genome-wide association results for fatty acid composition in Landrace. SNPs are plotted on the x-axis according to their position on each chromosome against statistical association with these traits on the y-axis. The *horizontal line* indicates genome-wide significance (*p* < 1.0^−6^)

**Fig. 3:**
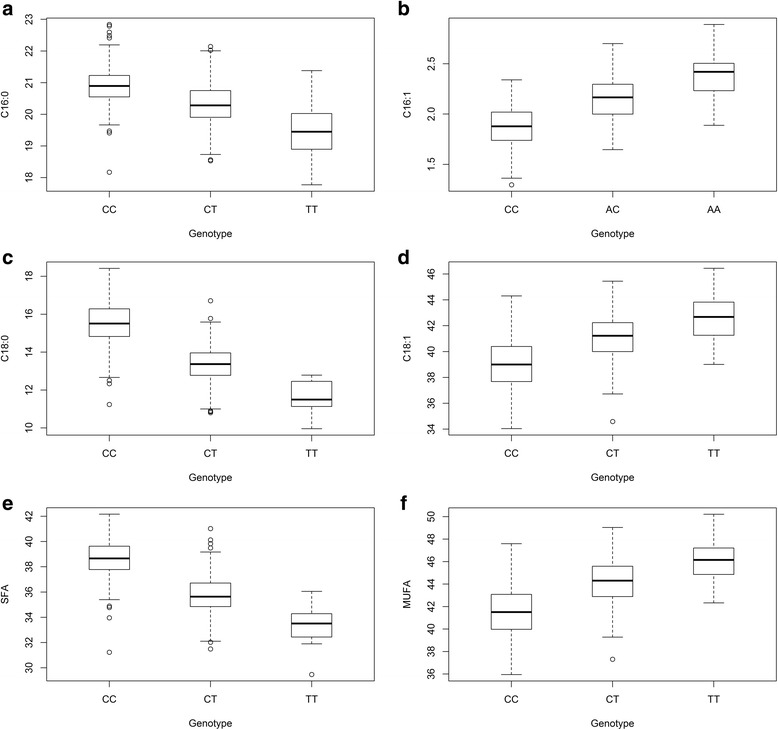
Phenotypic mean per genotype class for SSC14 SNPs in Duroc. Box plots showing the differences in levels of fatty acids for the different genotypes of the most significant SNP for each trait of significance (*rs318243431* for C16:0 and SFA, *rs340458768* for C16:1n-7 and *rs318695446* for C18:0, C18:1n-9 and MUFA). Box edges represent the upper and lower quartile with the median value shown as a bold line in the middle of the box. Whiskers represent 1.5 times the quartile of the data and individuals falling outside the range of the whiskers are shown as dots. **a** C16:0 **b** C16:1n-7 **c** C18:0 **d** C18:1n-9 **e** SFA **f** MUFA

A GWAS was conducted using the Axiom porcine 660 K array to identify loci associated with fatty acid composition in backfat of Duroc and Landrace boars. Descriptive statistics for the fatty acid phenotypes, summarized in Table 1, show that the two breeds differ with respect to fatty acid composition, with Landrace typically having higher levels of total MUFA, whereas Duroc on average have higher levels of total SFA [9]. The GWAS detect highly significant QTLs for the six *de novo* synthesized fatty acids (C16:0, C16:1n-7, C18:0, C18:1n-9, SFA and MUFA) on SSC4 and SSC14 in Duroc (Fig. 1 and Additional file 1). The QTL results are summarized in Table 2. The most significant results on SSC14 in Duroc were in the interval from 117.6 to 124.6 Mb. The most significant SNP for C16:0 and SFA was *rs318243431*, which is located at position 120,643,956 bp on SSC14 within intron 3 of the *carboxypeptidase N subunit 1* (*CPN1*) gene. The most significant SNP for C18:0, C18:1n-9 and MUFA was *rs318695446*, which is located at position 121,401,766 bp on SSC14, in an intergenic region between the genes *SCD* and *paired box 2* (*PAX2*). For C16:1n-7, the most significant SNP was *rs340458768*, which is located at SSC14 position 121,565,853 bp and falls within intron 8 of *PAX2*. There were 21 significant SNPs on SSC4 for all the *de novo* synthesized fatty acid traits and they are located at 63.85–63.99 Mb. In Landrace, a QTL on SSC8:120.5–121.4 Mb showed significant association for C16:1n-7 (Fig. 2, Table 2 and Additional file 2). The most significant SNP of this QTL is located in an intergenic region between two uncharacterized protein coding genes. No significant results were found for any of the other traits in Landrace. Moreover, no significant associations were found for the essential fatty acids (C18:2n-6, C18:3n-3 and total PUFA) in either Duroc or Landrace. Allele substitution effects and the proportion of explained genetic and phenotypic variance for the most significant SNPs are given in Table 3. On SSC14 in Duroc, the most significant SNP was shown to explain between 55 and 76% of the genetic variance and between 27 and 54% of the phenotypic variance for the different traits. For the most significant QTL in this study, region ~117–124 Mb on SSC14, box plots were made to visualize the mean phenotypic differences per genotype class for the most significant SNP (Fig. 3). The phenotypic difference is clear when comparing the three genotypes and indicates an additive genetic effect. Considering the human health perspective and a goal of reduced SFA and increased MUFA, the favorable allele for all traits in Duroc is the least frequent within the population.

**Table 1:** Summary statistics for fatty acid composition

Trait	Breed	n	Mean	SD	Min	Max
C16:0	Landrace	659	19.56	0.87	16.69	22.26
	Duroc	454	20.62	0.75	17.77	22.84
C16:1n-7	Landrace	659	2.40	0.20	1.52	3.08
	Duroc	454	2.03	0.26	1.30	2.89
C18:0	Landrace	659	11.61	1.00	8.83	16.98
	Duroc	454	14.49	1.64	9.96	18.42
C18:1n-9	Landrace	659	42.38	1.54	34.43	46.91
	Duroc	454	39.97	2.24	34.04	42.00
C18:2n-6	Landrace	659	15.90	1.74	10.71	23.61
	Duroc	454	15.69	1.61	10.95	20.64
C18:3n-3	Landrace	659	1.75	0.20	1.04	2.51
	Duroc	454	1.65	0.20	0.90	2.48
PUFA	Landrace	659	19.57	2.03	13.75	27.98
	Duroc	454	19.16	1.83	13.37	24.53
SFA	Landrace	659	33.54	1.55	29.58	40.07
	Duroc	454	37.29	2.20	29.47	42.17
MUFA	Landrace	659	45.92	1.67	36.52	50.77
	Duroc	454	42.79	2.60	35.95	50.22

For each breed, mean, standard deviation, minimum and maximum of the different fatty acids are presented (*n* = number of animals)

**Table 2:** QTL regions identified

SSC	Position (Mb)	Breed	Trait	#significant SNPs
4	63.85–63.99	Duroc	C16:0	21
		Duroc	C16:1n-7	21
		Duroc	C18:0	21
		Duroc	C18:1n-9	21
		Duroc	SFA	21
		Duroc	MUFA	21
8	120.5–121.4	Landrace	C16:1n-7	23
14	117.6–124.6	Duroc	C16:0	624
14	117.6–124.6	Duroc	C16:1n-7	648
	117.6–124.6	Duroc	C18:0	680
	118.1–123.5	Duroc	C18:1n-9	545
	117.6–124.6	Duroc	SFA	689
	118.1–123.5	Duroc	MUFA	566

Traits are listed with significant QTL regions (SSC and position (Mb)) and the number of significant SNPs within each QTL region (*p* < 1.0^−6^)

**Table 3:** The most significant SNP for each QTL and trait

SSC	Trait	Breed	SNP	MAF	Allele subst. effect	%σ^2^_a_	%σ^2^_p_	Significance (*p*-value)
4	C16:0	Duroc	*rs81241620*	0.22	0.60	51	25	6.22e-14
	C16:1n-7		*rs323595907*	0.22	−0.24	60	34	4.27e-14
	C18:0		*rs323595907*	0.22	1.82	67	48	3.40e-18
	C18:1n-9		*rs698347627*	0.22	−1.77	50	26	1.58e-11
	SFA		*rs323595907*	0.22	2.48	63	47	2.90e-19
	MUFA		*rs698347627*	0.22	−2.25	57	30	8.05e-13
8	C16:1n-7	Landrace	*rs324018164*	0.47	−0.06	19	5	3.48e-08
14	C16:0	Duroc	*rs318243431*	0.24	0.60	55	27	4.05e-15
	C16:1n-7		*rs340458768*	0.28	−0.25	71	40	5.83e-18
	C18:0		*rs318695446*	0.24	1.91	76	55	1.63e-21
	C18:1n-9		*rs318695446*	0.24	−1.82	55	28	2.00e-12
	SFA		*rs318243431*	0.24	2.55	71	54	3.16e-22
	MUFA		*rs318695446*	0.24	−2.31	62	33	2.76e-14

For each QTL region and trait analyzed with the 660 K array, the most significant SNP is presented with ID, minor allele frequency (MAF), allele substitution effect, proportion of explained genetic variation (%σ^2^_a_), proportion of explained phenotypic variation (%σ^2^_p_) and significance (*p*-value corrected for genomic control)

**Fig. 4:**
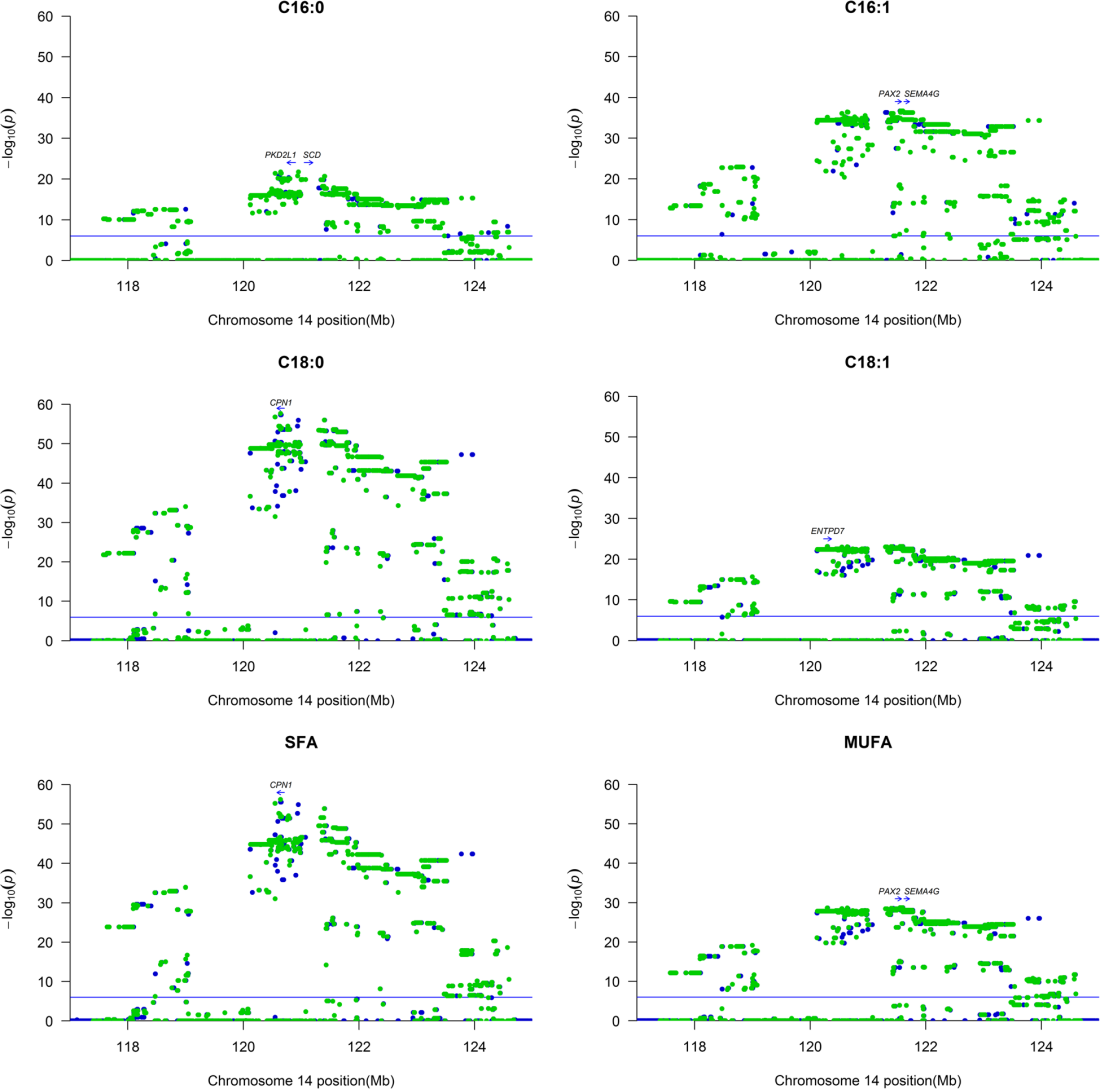
Association analyses using sequence variants within the QTL region on SSC14 in Duroc. *Green dots* are association results with imputed sequence variants; *blue dots* are GWAS results with 660 K SNPs. Genes located near the most significant SNP(s) are indicated with an *arrow* showing gene position and transcription direction. If the most significant SNP is in an intergenic region, the two surrounding genes are indicated

Whole genome re-sequencing data from Duroc (*n* = 23) boars was used to impute sequence-based genotypes in the 8 Mb QTL region on SSC14 in Duroc. The sequence based association study confirmed the findings of a highly significant QTL for the *de novo* synthesized fatty acids on SSC14 and revealed moderate to high LD in the QTL region at 120–122 Mb (Fig. 4). All significant SNPs in this region are given in Additional file 3. The most significant SNP for C16:0 was found at position 120,952,148 which is intergenic between the genes *polycystin 2 like 1, transient receptor potential cation channel* (*PKD2L1*) and *SCD*. For C16:1n-7 and MUFA, 21 highly significant SNPs, being in almost complete LD with each other, were located in the region at 121.56–121.60 Mb. The SNPs are positioned in introns and downstream of *PAX2*, and in the intergenic region between *PAX2* and the *semaphoring 4G* (*SEMAG4*) gene. The most significant SNP for C18:0 and SFA was at 120,643,956 Mb which is within intron 3 of *CPN1*. For C18:1n-9 the most significant SNPs, seven in total LD, were located at 120,29–120.30 Mb, which is in intron 5–7 of the gene *ectonucleoside triphosphate diphosphohydrolase 7* (*ENTPD7*).

The whole genome re-sequencing data was also used to examine the suggested causative variant *rs80912566* in the promotor of *SCD* [28]. While all three genotypes were found in the Duroc boars, the Landrace boars were fixed for the T allele, being the allele associated with higher fat desaturation. The LD between the *SCD* genotype and the most significant SNPs in Duroc *rs318695446*, *rs318243431* and *rs340458768* was *r* ^2^ = 0.82, 0.80 and 0.95, respectively. Based on findings of putative misplaced SNPs in a previous study [15], we also inspected the LD between the significant SNPs on SSC4 and SSC14, and found that the degree of LD between SNPs on these chromosomes was in the range of *r* ^2^ = 0.85–1.0.

## Discussion

Knowledge of the fatty acid composition of pork can be used to manipulate levels of different fatty acids and thereby produce pigs with healthier meat [2]. In the present study, we conducted GWAS for fatty acid composition in backfat from pigs of two different breeds regarding fat level and distribution [9]. This allowed us to identify genomic loci associated with levels of *de novo* synthesized fatty acids. For the essential fatty acids, C18:2n-6, C18:3n-3 and total PUFA, no QTLs were detected in either of the breeds, which is expected as the level of PUFA is influenced mainly by diet [1].

The most significant QTL in this study was identified on SSC14 in Duroc where it explains up to 76% of the genetic variance and 55% of the phenotypic variance for the *de novo* synthesized fatty acids. This is slightly higher compared with a previous study of the same QTL showing up to 45% explained by genetic variance [25]. The QTL region from the GWAS is at 117.6–124.6 Mb and the fine mapping using sequence data narrowed down the peak to region 120–122 Mb. This QTL region has also previously been identified in subcutaneous fat and intramuscular fat of Duroc pigs at 120–124 Mb [16, 25] and in the *longissimus dorsi* muscle of different breeds and crossbreds [15, 20, 21]. *SCD*, located at 120.96–120.98 Mb and encoding the main enzyme responsible for the desaturation of SFA to MUFA, is considered a very strong positional candidate gene for this QTL. *SCD* has been investigated as a candidate gene underlying fatty acid composition in pigs in several studies [15, 16, 25, 28–30] and different haplotypes with *SCD* variants have been found to be significant [30, 32]. After studying SNPs and haplotypes of *SCD* in different breeds, Estany and co-authors [28] suggested that the *rs80912566* SNP, identified in Uemoto and co-authors [32], within the promoter region is the putative causative variant. This SNP is positioned in the core sequence of several putative transcription factor binding sites and was included in our fine mapping using sequence data. Results showed that it was among the highly associated SNPs but not the most significant one for either of the traits analyzed. The LD between *rs80912566* and the most significant SNPs detected by the GWAS was *r* ^2^ = 0.80–0.95, so our most significant SNPs would pick up the signal of *rs80912566* if it is indeed the causal variant. We also examined the effect of the different homozygote genotypes of *rs80912566* on the actual phenotypic level of fatty acids, as done in Fig. 3 by the top SNPs. The difference between AA and BB for levels of C16:0, expressed as % of total fatty acids, was 1.16 for *rs80912566* and 1.43 for *rs318243431*. For C16:1, the difference was 0.5 for *rs80912566* and 0.51 for *rs340458768*. Based on such marginal differences between our most significant SNP and the *rs80912566 (SCD)* we were not able to conclude which is the most likely causal variant.

Due to high LD in the QTL region on SSC14, it is impossible to conclude with specific causative genes or SNPs merely on fine mapping results. Positional candidate genes suggested from the GWAS positions obtained by 660 K and/or sequence data are *SCD*, *CPN1*, *PAX2*, *PKD2L1*, *ENTPD7* and *SEMA4G*. The functions of *PAX2*, *ENTPD7* and *SEMA4G* do not support a role in fatty acid composition, but the three other genes might be considered as biological interesting candidates. The protein encoded by *CPN1* is the small subunit of carboxypeptidase N, which is a metalloprotease that regulates peptide activity and receptor binding [55]. The carboxypeptidase member E protein expression has been linked to fatty acid levels in human [56], however, the biological function of carboxypeptidase member CPN1 is not fully understood [55]. *PKD2L1* belongs to the polycystin family of transient receptor potential channel superfamily and it has previously been associated with levels of C16:1n-7 [57, 58] and the phospholipid C16:0 to C18:0 ratio [58]. However, the exact role of *PKD2L1* is not known and the study by Wu et al. 2013 [57] suggested that *SCD* was a better candidate gene due to its function. The thorough characterization of *SCD* and its localization in the QTL peak makes it a very strong candidate gene for the association, however, from our results we cannot conclude that it is the causal one.

On SSC4, 21 significant SNPs were associated with the *de novo* synthesized fatty acids in Duroc at 63.85–63.99 Mb. A QTL for fatty acid composition has previously been identified close to this QTL region, at ~60 Mb [17, 27]. The SNP *INRA0046679* at 63.8 Mb on SSC4 in a study by Yang et al. [15] was significant for C18:0, however, the SNP was in complete LD with the most significant SNP on SSC14 (~121 Mb) and the authors therefore concluded that this SNP is misplaced. In this study, the SNPs on SSC4 displayed high or complete LD (*r* ^2^ = 0.85–1.0) with the most significant SNPs on SSC14. We therefore suspect, as was concluded in the study by Yang et al. [15], that the SNPs are misplaced on the marker map, probably due to errors in the reference genome [59].

In Landrace, we identified a QTL for C16:1n-7 on SSC8 with significant SNPs located between 120.5 and 121.4 Mb. Several previous studies have reported QTLs for fatty acid composition on this chromosome [13, 16–18, 21, 27]. One GWAS obtained a QTL overlapping the one we identified for the fatty acids C14:0, C16:1n-7 and C20:3 in backfat, as well as for C16:0 and C16:1n-7 in intramuscular fat [17]. The authors also identified genes surrounding this QTL region through eQTL analysis and the candidate gene closest to our most significant SNPs is *ELOVL6*, located at 120.1–120.2 Mb. This gene encodes a fatty acid elongase that is involved in the elongation of C12-16 fatty acids to C18 [60]. Studies investigating polymorphisms in *ELOVL6* in pig identified promoter SNPs associated with C16:0 and C16:1n-7 in backfat [27, 61] and suggested that a SNP located at −394 bp from the transcription start is a potential causative mutation [61]. This SNP is associated with an increased methylation level of the *ELOVL6* promoter and decreased gene expression. The promoter SNP was found to explain 32% of the phenotypic variance for C16 in backfat of Iberian x Landrace pigs [61] whereas the most significant SNP in our study, *rs324018164*, explained 19% of the genetic variance and 5% of the phenotypic variance for C16:1n-7. Whether the *ELOVL6* promoter SNP is the causative variant in Landrace needs to be further investigated.

It has been suggested that having high levels of C18:1n-9 in meat is favorable for technological quality and sensory properties, in addition to being favorable for human nutrition [1]. Fat from commercial pigs already contains 40–45% C18:1n-9, which is either *de novo* synthesized from carbohydrates in the diet or comes directly from the diet. Breeding for more C18:1n-9 is possible since the trait is highly heritable (h^2^ = 0.60) [9, 35], and now also by use of the QTL detected in this study. As shown in Fig. 3 the average phenotypic level will change from 39% C18:1n-9 for the TT genotype of *rs318695446*, to 41% for TC and 42.5% for CC, and the C18:0 will decrease accordingly. In general, oleic acid content is not a valued trait by meat producers and, consequently, not many breeding companies are focusing on fatty acid composition. However, it is an interesting trait in the particular case of traditional dry-cured ham and healthier pork products, and some breeding companies are exploring selecting for this trait.

## Conclusions

The present study confirms the importance of previously identified QTLs for fatty acid composition on SSC14 in Duroc pigs and on SSC8 in Landrace pigs. The most significant SNP in this study was found for Duroc on SSC14 and explained between 55 and 76% of the genetic variance and between 27 and 54% of the phenotypic variance for the *de novo* synthesized fatty acid traits. Fine mapping of the QTL region on SSC14 confirmed the QTL but high LD made it difficult to identify causative variants. Based on our results the putative functional SNP suggested within *SCD* could not be proven to be the causal one. In Landrace, a significant QTL was identified on SSC8 for C16:1n-7, explaining 19% of the genetic variance and 5% of the phenotypic variance. The results of this study can be implemented in breeding to produce higher quality and healthier fatty acid composition of pork meat.

## Additional files

Additional file 1: Significant association statistics for the fatty acid traits in Duroc using the 660 K SNP array. For each trait, the significant SNPs are presented with SNP ID, chromosome and basepair position, and *p*-value. (XLSX 5879 kb)

Additional file 2: Significant association statistics for the fatty acid traits in Landrace using the 660 K SNP array. For each trait, the significant SNPs are presented with SNP ID, chromosome and basepair position, and *p*-value. (XLSX 10 kb)

Additional file 3: Significant association statistics for the imputed sequence variants in the QTL region on SSC14 in Duroc. SNPs are presented with ID, chromosome, position and multiple testing adjusted *p*-values. (XLSX 297 kb)

## Abbreviations

AI: Artificial insemination
CPN1: Carboxypeptidase N subunit 1
ELOVL6: ELOVL fatty acid elongase 6
ENTPD7: Ectonucleoside triphosphate diphosphohydrolase 7
FASTA: Family Based Score Test for Association
GLM: Generalized linear model
GWAS: Genome-wide association study
Indel: Insertion/deletion
LD: Linkage disequilibrium
MAF: Minor allele frequency
MTTP: Microsomal triglyceride transfer protein
MUFA: Monounsaturated fatty acids
NIRS: Near-infrared spectroscopy
PAX2: Paired box 2
PKD2L1: Polycystin 2 like 1, transient receptor potential cation channel
PUFA: Polyunsaturated fatty acids
QTL: Quantitative trait loci
SCD: Stearoyl-CoA desaturase
SEMA4G: Semaphoring 4G
SFA: Saturated fatty acids
SNP: Single nucleotide polymorphism
VEP: Variant Effect Predictor
